# Experimental Biomechanical study on the consequences of dashboard injury of the pelvis after total hip replacement

**DOI:** 10.1007/s00414-025-03633-9

**Published:** 2025-10-07

**Authors:** Kálmán Rácz, Gábor Simon, Gyula Győrfi, László Kiss, Tamás Bazsó, Loránd Csámer, Tamás Juhász, Péter Attila Gergely, Sándor Manó

**Affiliations:** 1 Department of Forensic Medicine, Faculty of Medicine, University of Debrecen, Nagyerdei krt. 98, Debrecen, H-4032 Hungary; 2 Department of Forensic Medicine, Medical School, University of Pécs, 12 Szigeti street, Pécs, H-7624 Hungary; 3 Department of Orthopaedic Surgery, Faculty of Medicine, University of Debrecen, Nagyerdei krt. 98, Debrecen, H-4032 Hungary; 4 Laboratory of Biomechanics, Innovation Ecosystem Centre, University of Debrecen, Vezér u. 37, Debrecen, H-4032 Hungary; 5 Department of Anatomy, Histology and Embryology, Faculty of Medicine, University of Debrecen, Nagyerdei krt. 98, Debrecen, H-4032 Hungary; 6 Department of Mechanical Engineering, Faculty of Engineering, University of Debrecen, Ótemető u. 2-4, Debrecen, H-4028 Hungary

**Keywords:** Forensic pathology, Total hip replacement (THR), Periprosthetic fracture, Traffic accident, Dashboard injury

## Abstract

Fractures of the pelvic ring and acetabulum are caused by high-energy trauma, usually by traffic accidents. Acetabular fractures occur when a force drives the head of the femur against the acetabulum. As the prevalence of patients living with a total hip prosthesis (THR) increases, the chance of suffering periprosthetic acetabulum fractures also elevates. However, the injury threshold of forces resulting in a periprosthetic acetabular fracture is unknown. The study aimed to analyze the results of a dashboard injury on the acetabulum after total hip replacement through a head-on collision in an ex-vivo experiment. A cemented cup was implanted into hemipelves removed from cadavers, and a dashboard injury was simulated with an impact of a pendulum-like structure released from different heights. The impact energy increased until inflicting acetabular fracture. Eleven hemipelves were examined, of which five were male and six were female. The average force required to cause damage to the pelvis was 5852 N (3950–8386). Isolated acetabular component loosening was noticed with cement fracture in one case (at 5344 N force), acetabular cup loosening occurred combined with posterior column fracture in three cases (at 8386, 3950, 6295 N force), and acetabular cup loosening combined with acetabular floor fracture occurred in six cases (at 4305, 4573, 6531, 4707, 8174, 6117 N force). A combination of all three mechanisms occurred in one case: acetabular cup loosening, with posterior column and acetabular floor fracture at 5986 N force. The results of the ex-vivo experiment indicate that in a dashboard injury, at least around 4000 N force and 4 J impact energy is necessary to create a periacetabular fracture. The results suggest that a larger force is necessary for damage to occur in male pelvises: fractures occurred mostly below 5000 N force in female pelvises, while they occurred above 6000 N in most males.

## Introduction

Pelvic fracture constitutes around 3% of overall fractures, but it is associated with high mortality [1] and a high complication rate and often results in inadequate functional outcome [2, 3]. Fractures to the pelvic ring and acetabulum are caused by high-energy trauma, usually by traffic accidents [4]. Approximately 1.19 million people die each year as a result of a traffic accident [5], and 25% of them suffer pelvic fractures [6]. However, the incidence of pelvic fractures is greatly influenced by vehicle safety [7].

Acetabular fractures occur when a force drives the head of the femur against the acetabulum. A typical mechanism is the dashboard injury, where the direct blow to the anterior aspect of the knee due to its impact on the dashboard pushes the femur backward [8]. According to cadaveric experiments, approximately half of the peak force applied to the knee is transmitted to the hip in these types of accidents [9]. Other mechanisms of injury include direct blows to the lateral aspect of the hip during side impact car collisions or falling from height [8]. The role of brake pedal injury in the development of hip injuries also has to be considered as an important mechanism [10, 11].

The acetabulum fractures can be subcategorized, the most common being due to the sitting position with the hips and knees flexed 90 degrees, the postero-superior hip dislocation with posterior acetabular column fracture, or in case the leg was in abduction central hip dislocation with acetabulum floor fracture can occur. It is well documented that during dashboard injury by impact through the flexed knee, due to the force acting longitudinally on the femur, injuries can occur anywhere between the knee and the acetabulum [8, 9, 12].

The number of total hip replacements (THR) performed is rising yearly, reaching more than 300/100.000 population in some developed countries [13–16], so the prevalence of patients living with a THR is increasing. Since most of the patients who underwent THR want to live an active life, including the demand to drive a motor vehicle, the chance of them suffering a traffic accident is rising every year. Several other types of injuries may occur in patients after THR: periprosthetic fractures on the femur [17–19] or any part of the acetabulum [20, 21], loosening of one or more components, hip dislocation [12, 22] or several combinations of these. Managing periprosthetic fractures is challenging, and these injuries generally have unfavorable outcomes [23]. Periprosthetic acetabulum fractures are rare but occur more frequently due to the increase in patients who underwent THR [24]. Except for a few cases, these injuries require surgery, with weeks of hospitalization and extended rehabilitation. However, scientific literature about these types of fractures is scarce [24]. The factors determining the type of injury, whether it depends on the amount of energy absorbed, the position of the lower limb or the direction of the impacting force, remain unclear. The injury threshold of forces resulting in a periprosthetic acetabular fracture is also unknown.

This study aimed to analyze the results of a dashboard injury on the acetabulum after total hip replacement through a head-on collision in an ex-vivo experiment.

## Materials and methods

The study and publishing the results was approved by the Regional Research Ethic Committee, Debrecen (DE OEC RKEB/IKEB 3312 − 2011.)

Eleven hemipelvis were removed from the cadavers deceased due to non-traumatic causes and had no pelvic fracture in their medical history. Five cadavers were male, and six were female, with an average age of 68.3 (52–98) years (Table 1). The specimens were removed during the autopsy of the deceased. Body height and body weight were registered prior to the autopsy. The specimens were removed with the help of a mediolateral and horizontal incision in the hip region. After removing the external muscles (gluteal and hip adductors) and internal muscles (iliopsoas group) from the pelvic bone, the femur was separated from the acetabulum by dissecting the hip ligaments. The hemipelvis was removed from the cadaver by transecting the symphysis and the sacroiliac joint. The maximum acetabulum diameter was measured with a digital vernier caliper (Workzone, accuracy: 0.01 m) in a superior to the inferior direction (Table 1). Prior to the experiment, hemipelvis specimens were stored at 4 C. The experiments were performed within 24 h after specimen removal. A cemented cup (Sanatmetal UHMW polyethylene cemented cup) was implanted into all hemipelvis with 45 degrees of abduction and 10 degrees ante-version.

**Table 1:** Caracteristics of the cadavers

No.	1	2	3	4	5	6	7	8	9	10	11
Sex (M/F)	M	F	M	F	F	M	F	F	M	F	M
Age (years)	52	59	60	62	64	64	69	73	73	77	98
Height (cm)	180	167	181	176	164	178	161	169	178	156	165
Weight (kg)	78	53	82	71	51	75	49	67	77	54	62
AD (mm)	62	56	62	57	55	61	50	56	63	49	53

AD: maximum acetabulum diameter is measured in a superior to inferior direction

**Fig. 1:**
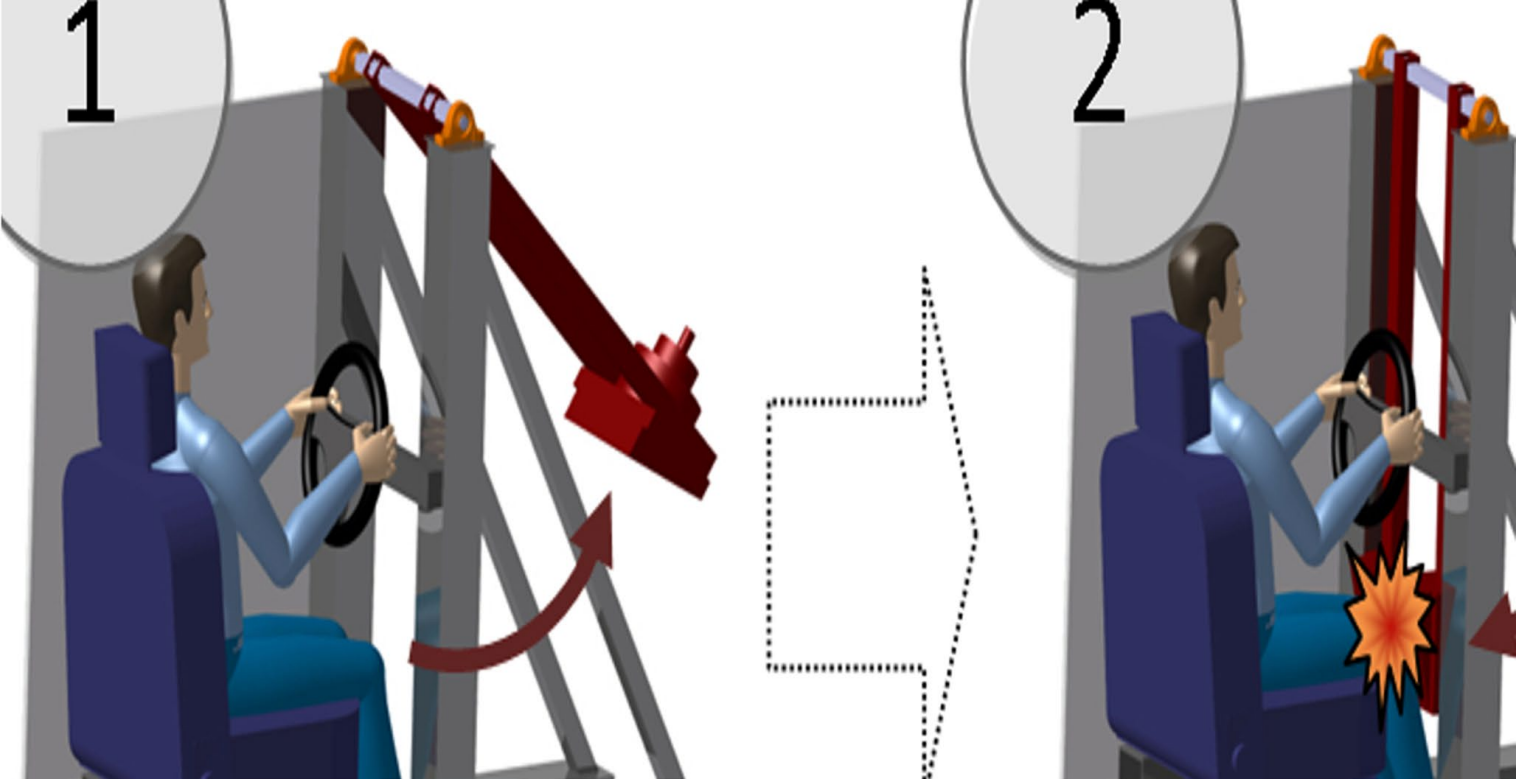
Schematics of the hitting frame simulating dashboard

**Fig. 2:**
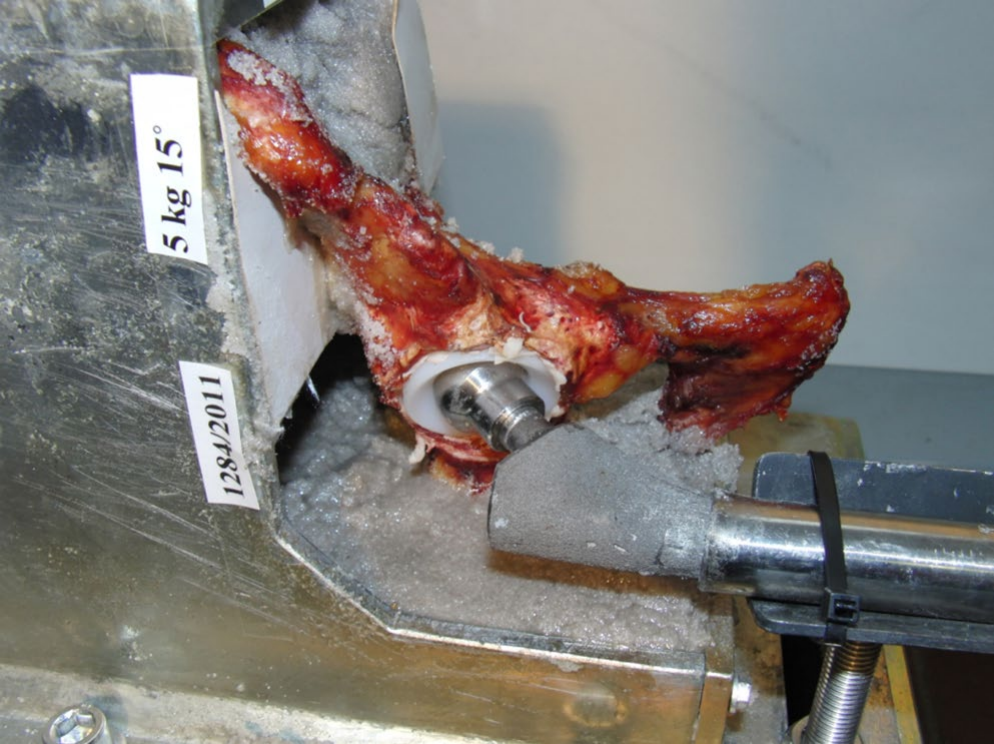
The experimental set up

A hitting frame simulating dashboard injury was developed (Fig. 1). The eleven hemipelvis were fixed with epoxy resin (KEMAPOX Grund 2000, Murexin) to the hitting frame. The femur was simulated with a metal rod that had the proximal part of a femoral component of the prothesis fixed to it to articulate with the cemented acetabular cup (Fig. 2.). To the vertical part of the frame, a pendulum-like structure was fixed that was released from different heights to impact the distal end of the metal rod simulating the femur. The impact occurred in the neutral position of the hip without ab- or adduction. The force of impact could be changed by increasing the angle and weight. The distal end was fitted with a 2.5 kN load cell (Dynacell, Instron Ltd, High Wycombe, UK) which recorded the force of impact through Instron Max software (Instron Ltd, High Wycombe, UK).

The fixed hemipelvis was examined macroscopically for damage after each impact. The impact energy was increased until visible damage was achieved. The impact energy was increased by increasing the angle the pendulum was dropped from. The angles used were 15, 22, 30, 37, 45 and 52 degrees. If no damage occurred at the largest angle, then the weight of the pendulum was increased. The weight of the pendulum was 15 kg in one case, and 5 kg in all other cases. The angle and height of the pendulum upon release were noted, along with the force of impact required for damage to occur.

## Results

The average force required to cause damage to the pelvis was 5852 N (3950–8386). The smallest angle at which the pendulum was released for damage to occur was 22 degrees, and the largest was 52 degrees. The pendulum release height in these cases was 0.08 m and 0.32 m (Table 2). The loading and unloading of the prosthesis occurred in 15 ms on average.

**Table 2:** Results of the measurements

No.	Sex (M/F)	Age (years)	Experiment (no)	Pendulum weight (kg)	Swing angle (°)	Swing height (m)	Energy (J)	Max. Force (*N*)	Fracture (Y/*N*)
1	M	52	1/1	5	15	0,037	1,81	3249	no
			1/2	5	22	0,08	3.92	3670	no
			1/3	5	37	0.222	10.89	6117	yes
2	F	59	2/1	5	15	0,037	1,81	3473	no
			2/2	5	22	0,08	3.92	5227	no
			2/3	5	37	0.222	10.89	6295	yes
3	M	60	3/1	5	15	0,037	1,81	2731	no
			3/2	5	45	0,322	15.79	6838	no
			3/3	15	30	0,147	21,63	8550	no
			3/4	15	45	0,322	47.38	8386	yes
4	F	62	4/1	5	15	0,037	1,81	3474	no
			4/2	5	22	0,08	3.92	3750	no
			4/3	5	30	0,147	7.21	4278	no
			4/4	5	37	0.222	10.89	4707	yes
5	F	64	5/1	5	15	0,037	1,81	3712	no
			5/2	5	22	0,08	3.92	3958	no
			5/3	5	37	0.222	10.89	5122	no
			5/4	5	45	0,322	15.79	5986	yes
6	M	64	6/1	5	15	0,037	1,81	2826	no
			6/2	5	22	0,08	3.92	5344	yes
7	F	69	7/1	5	15	0,037	1,81	2901	no
			7/2	5	22	0,08	3.92	3822	no
			7/3	5	30	0,147	7.21	4305	yes
8	F	73	8/1	5	15	0,037	1,81	2453	no
			8/4	5	30	0,147	7.21	4573	yes
9	M	73	9/1	5	15	0,037	1,81	2851	no
			9/2	5	37	0.222	10.89	6884	no
			9/3	5	45	0,322	15.79	8957	no
			9/4	5	52	0,423	20.75	8174	yes
10	F	77	10/1	5	15	0,037	1,81	2119	no
			10/2	5	22	0,08	3.92	3950	yes
11	M	98	11/1	5	30	0,147	7.21	4043	no
			11/2	5	45	0,322	15.79	6531	yes

**Fig. 3:**
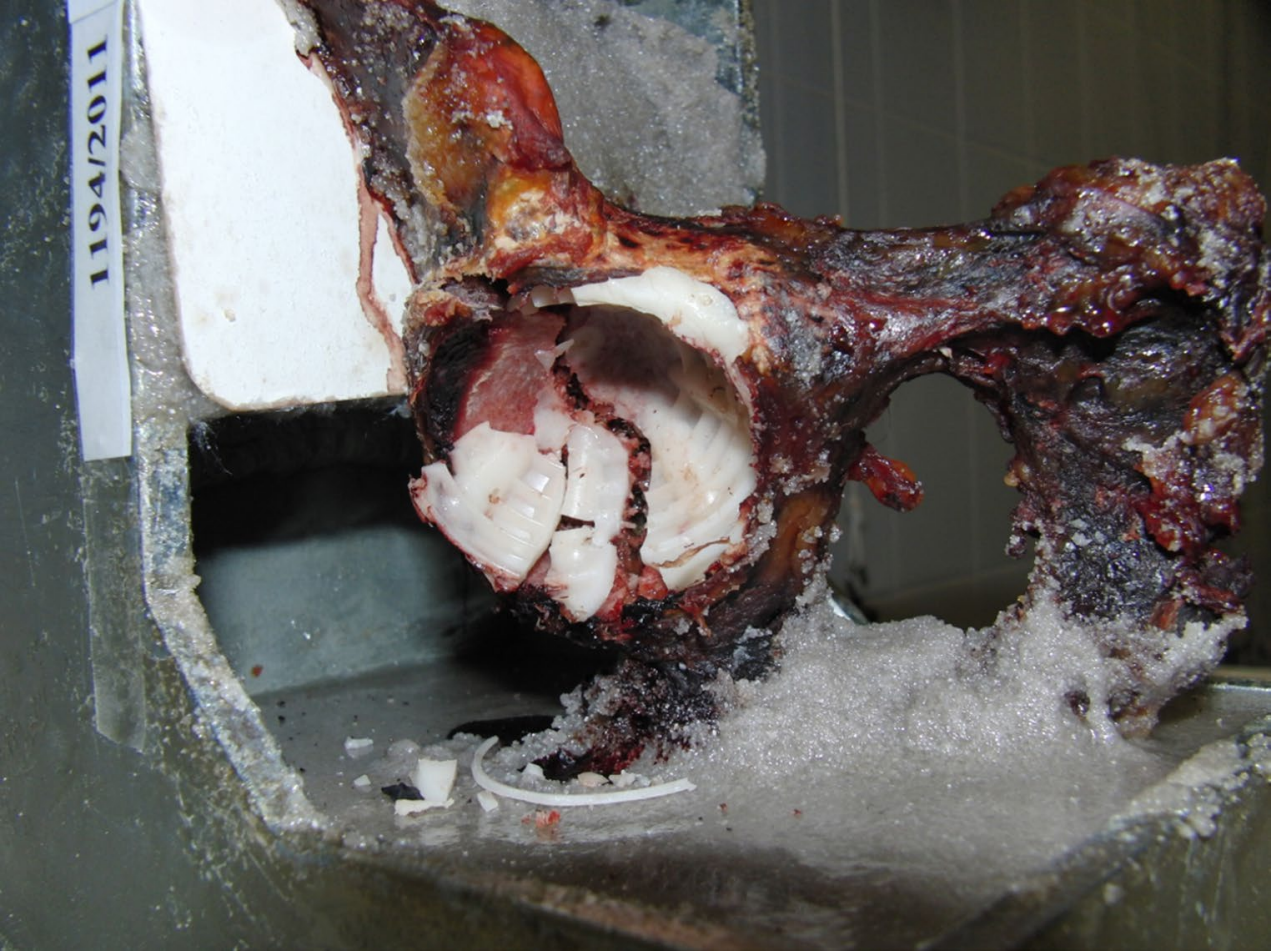
Acetabular cup loosening, with posterior column and acetabular floor fracture

The following mechanisms of injury occurred: isolated acetabular component loosening was noticed with cement fracture in one case (at 5344 N force), acetabular cup loosening occurred combined with posterior column fracture in three cases (at 8386, 3950, 6295 N force), and acetabular cup loosening combined with acetabular floor fracture occurred in six cases (at 4305, 4573, 6531, 4707, 8174, 6117 N force). A combination of all three mechanisms occurred in one case, which is acetabular cup loosening, with posterior column and acetabular floor fracture at 5986 N force (Fig. 3).

In the case of two hemipelvis, the maximal recorded energy at the last impact (3/4 and 9/4) was lower than in the case of the previous impact (no. 3/3 and 3/4), which could be explained theoretically that a microfracture invisible macroscopically weakened the bone in the previous impacts (3/3 and 9/3).

## Discussion

Several factors influence the occurrence of acetabular and periprosthetic acetabular fractures. Apart from the mechanism (direction of impact, position of femur) and the impact energy, individual factors such as sex, age and osteoporosis also have to be considered. The elasticity of the soft tissues and rigidity of the pelvis also influence acetabulum [25] injuries by affecting the energy structures absorb, and the energy absorption potential of the seat is also significant.

Biomechanical studies of fixation methods in acetabular fractures has been previously performed. However, these studies were aimed at evaluating and comparing the different fixation methods applicable in the case of acetabulum fractures under physiological loading [26]. Biomechanical studies are also available about the stability of the implanted acetabular cup [27, 28], but no data is available about the consequences of forces representing high-energy impacts.

The results of our study indicate that in case of dashboard injury, at least around 4000 N force and 4 J impact energy are necessary to create a periacetabular fracture so that these forces can be used as a threshold for injury. Analyzing the results based on gender revealed that a 40% larger force was required for damage to occur in male pelvises. Fractures occurred mostly below 5000 N force in female pelvises, while they occurred above 6000 N in most males. The results suggested that the force required for a fracture decreased with age in female pelvises, but a definite statement on the effect of the age on the threshold of fracture can not be made due to the low number of cases.

It also has to be emphasized, however, that the injury threshold in real-life accidents can be higher since the effect of the musculature, ligaments and connective tissue formation around the replaced hip that occurs in vivo but was not reproducible in our ex vivo experiment also affects the absorbed energy and biomechanical response of the replaced hip to a trauma. The energy absorption potential of external factors (e.g. seat) also has to be considered in a real-life accident.

It is important to emphasize that if trauma occurring in the acetabular region due to deceleration is compared between individuals with and without hip replacement, those with replaced hips are at a greater disadvantage regarding the consequences of the injury. With the exception of isolated hip dislocation, all other injuries occurring in patients with replaced hips due to dashboard injury require surgery [20]. The reconstruction of these hips is technically challenging due to the bone loss and the risk of infection is also increased. Since these accidents often occur in still active, working individuals, the extended rehabilitation following surgical reconstruction can cause severe problems in the lives of these people.

Obtaining more data about the biomechanical behavior of prothetised pelvises in high-energy impacts and determining the threshold of injury will help forensic evaluation of this injury type and also may help to avoid injuries that occurred during our experiment by using the results for improving the safety of vehicles.

## Limitations

The study was only performed on a few specimens; therefore, no statistical analysis could be performed. The average age of the cadavers was 68 years, but that represents well that total hip replacements are mainly installed in the elderly. However, because of the large individual differences in bone strength, especially as osteoporosis affects the given age group extensively, a study involving a larger number of specimens would be needed to draw a more definite conclusion. After performing a well-founded statistical analysis, the bone density measurements must also be included in future studies.

It has to be considered an important limitation that the specimens were exposed to multiple impacts before a visible fracture occurred. It is possible, however, that previous impacts also caused structural injury (microfractures not visible by observation), which could weaken the bone before the next impact. The structural integrity of the specimens should be checked (e.g., with micro-CT) before each impact in future experiments.

During the examination, the hip was in a neutral position, as slight abduction or adduction of the hip can significantly change the mechanism of impact. These different impact mechanisms also have to be examined in the future.

The role of the whole pelvic ring and the soft tissues in the absorption and dissipation of the load is significant and, therefore, can increase the threshold of force which could create a fracture. Due to ethical limitations, only hemipelvis could be used in this study, so future experiments involving these structures would be necessary if ethical permission could be obtained.

## Conclusion

At least around 4000 N force and 4 J impact energy are necessary to create a periacetabular fracture during a dashboard injury to use these forces as a threshold for injury. However, further studies are necessary to analyze the effect of individual differences.

## Data Availability

The data is included in the manuscript in Tables 1 and 2. Furthermore, the raw data from the experiment (.xls files with time, displacement and force) is available from authors upon request.
